# Supplementary material for: Preparation of Cellulose-Grafted Acrylic Acid Stabilized Jujube Branch Biochar-Supported Nano Zero-Valent Iron Composite for Cr(VI) Removal from Water
Source: Nanomaterials (Basel). 2025 Mar 14;15(6):441. doi: 10.3390/nano15060441 (PMC11944448; doi:10.3390/nano15060441)
Supplement: Supplementary file 1 [file nanomaterials-15-00441-s001.zip › nanomaterials-3523011-supplementary.pdf]

Supporting Information

PPreparation of Cellulose-Grafted Acrylic Acid Stabilized Jujube Branch Biochar-Supported Nano Zero-Valent Iron Composite for Cr(VI) Removal from Water

Xiaoxue Wang 1, Zhe Tan 1, Shuang Shi 1, Shanyuan Zhang 1, Shuang Yang 1, Xingyu Zhang 2, Pingqiang Gao 1,3,* and Yan Zhang 1,*

1 School of Chemistry and Chemical Engineering, Yulin University, No. 51 Chongwen Road, Yulin 719000, China

2 School of Materials Science and Engineering, Xi'an University of Technology, No. 5 Jinhua South Road, Beilin District, Xi'an 710048, China

3 Yulin Engineering Research Center of Coal Chemical Wastewater, Yulin University, No. 51 Chongwen Road, Yulin 719000, China

* *Correspondence: gpqzy2013@sina.com (P.G.); zhangyan@yulinu.edu.cn (Y.Z.); Tel./Fax: +86-0912-3282878 (P.G. & Y.Z.)*

Supporting figures

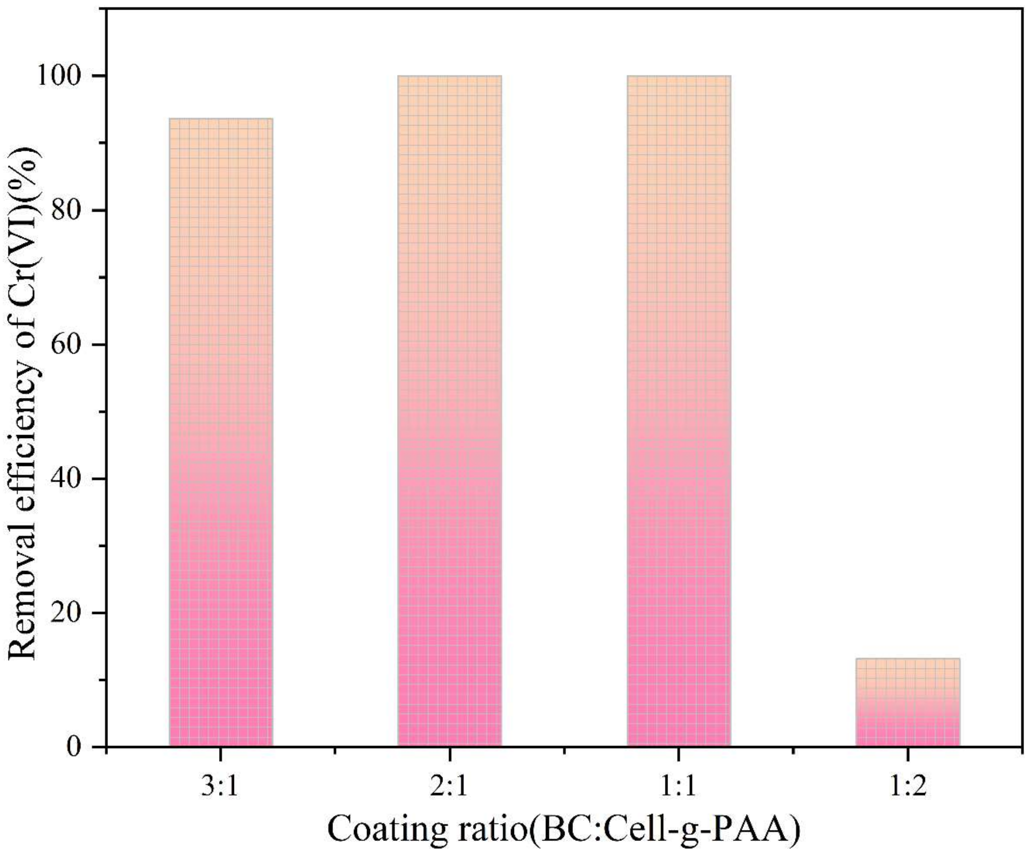

Figure S1. Effect of coating ratio on Cr(VI) removal.
